# T-CLEARE: a pilot community-driven tissue clearing protocol repository

**DOI:** 10.3389/fbioe.2024.1304622

**Published:** 2024-09-16

**Authors:** Kurt R. Weiss, Jan Huisken, Neda Khanjani, Vesselina Bakalov, Michelle L. Engle, Michelle C. Krzyzanowski, Tom Madden, Deborah R. Maiese, Justin R. Waterfield, David N. Williams, Lauren Wood, Xin Wu, Carol M. Hamilton, Wayne Huggins

**Affiliations:** ^1^ Morgridge Institute for Research, Madison, WI, United States; ^2^ Mark and Mary Stevens Neuroimaging and Informatics Institute, Laboratory of Neuro Imaging, Keck School of Medicine of University of Southern California, Los Angeles, CA, United States; ^3^ Bioinformatics and Computational Biology Program, RTI International, Durham, NC, United States

**Keywords:** microscopy, tissue clearing, tissue clearing methods, database, neuroscience

## Abstract

Selecting and implementing a tissue clearing protocol is challenging. Established more than 100 years ago, tissue clearing is still a rapidly evolving field of research. There are currently many published protocols to choose from, and each performs better or worse across a range of key evaluation factors (e.g., speed, cost, tissue stability, fluorescence quenching). Additionally, tissue clearing protocols are often optimized for specific experimental contexts, and applying an existing protocol to a new problem can require a lengthy period of adaptation by trial and error. Although the primary literature and review articles provide a useful starting point for optimization, there is growing recognition that results can vary dramatically with changes to tissue type or antibody used. To help address this issue, we have developed a novel, freely available repository of tissue clearing protocols named T-CLEARE (Tissue CLEAring protocol REpository; https://doryworkspace.org/doryviz). T-CLEARE incorporates community responses to an open survey designed to capture details not commonly found in the scientific literature, including modifications to published protocols required for specific use cases and instances when tissue clearing protocols did not perform well (negative results). The goal of T-CLEARE is to help the community share evaluations and modifications of tissue clearing protocols for various tissue types and potentially identify best-in-class methods for a given application.

## Introduction

Tissue clearing refers to methods that increase the physical transparency of biological samples. Through a series of chemical or physical steps, tissue clearing methods homogenize the refractive index of a sample by removing, replacing, or modifying light-scattering components (e.g., lipids and water). This process increases light transmission and optical imaging depth, making it possible to capture volumetric images of large samples without sectioning the material into thin slices. In recent years, tissue clearing has made it possible to generate high-resolution, three-dimensional (3D) images of entire brains, peripheral organs, bones, and embryos from model organisms (Dodt et al., 2007; Keller et al., 2008; Ahrens et al., 2013; Susaki et al., 2014; Vladimirov et al., 2014; Chhetri et al., 2015; Lemon et al., 2015; Tomer et al., 2015; Stefaniuk et al., 2016; Fretaud et al., 2017; Greenbaum et al., 2017; Affaticati et al., 2018; Mzinza et al., 2018; Xu et al., 2019; BRAIN Initiative Cell Census Network, 2020; Kirchner et al., 2020; Woo et al., 2020; Moatti et al., 2022), and large, intact sections of human tissue including brain, breast, prostate, and pancreas (Chung et al., 2013; Liebmann et al., 2016; van Royen et al., 2016; Noe et al., 2018). Data from these images have been used for developmental biology (Vieites-Prado and Renier, 2021), cellular phenotyping (Yang et al., 2014; Gerfen et al., 2016), identifying and characterizing brain regions governing behavior (Renier et al., 2016; Ye et al., 2016; BRAIN Initiative Cell Census Network, 2021), and defining structural aspects of disease pathologies (Liebmann et al., 2016; van Royen et al., 2016; Hong et al., 2019; Sabdyusheva Litschauer et al., 2020).

Selecting and implementing a tissue clearing protocol can be challenging. Several excellent reviews highlight that no single tissue clearing protocol (or subset of protocols) is broadly applicable across different experimental contexts. Instead, there are many published tissue clearing techniques, and each has its strengths and weaknesses (e.g., cost, speed, labeling strategy, toxicity, tissue morphology preservation, and instrument compatibility) (Richardson and Lichtman, 2015; Azaripour et al., 2016; Seo et al., 2016; Tainaka et al., 2016; Ariel, 2017; Tainaka et al., 2018; Yu et al., 2018; Costantini et al., 2019; Ueda et al., 2020; Richardson et al., 2021; Weiss and Huisken, 2021). Thus, the first step for selecting a protocol is to review the literature for similar experiments in similar tissues. Once chosen, the tissue clearing strategy often must be adapted for specific experimental parameters and research goals (Weiss and Huisken, 2021). Published articles and protocol repositories, such as protocols.io, usually report protocols after they have been optimized and do not provide lessons learned and modifications that were required to generate a successful result. Online forums (e.g., https://forum.image.sc/) provide valuable opportunities to solicit advice from experienced researchers and protocol developers, but unstructured text can complicate searching and compiling related content across posts.

This article describes the development and implementation of T-CLEARE (Tissue CLEAring protocol REpository; https://doryworkspace.org/doryviz). T-CLEARE is intended to complement publications, protocol repositories and forums and help the community more effectively compare and evaluate protocols when designing a tissue clearing strategy. T-CLEARE is based on the results of a survey that asked researchers to identify tissue clearing protocols they have used. Respondents were also asked to provide details not usually found in publications or protocol repositories, including protocol effectiveness and ease of use, modifications needed to optimize a protocol for specific circumstances, pre- and postprocessing steps not specific to published protocols, and negative results (i.e., instances when a tissue clearing protocol did not perform as expected or was not appropriate for the application). T-CLEARE represents each tissue clearing protocol reported by the community as a flow chart of five steps: fixation, pretreatment/decolorization, labeling, delipidation, and refractive index matching. Each flow chart is included in a tree diagram and organized according to whether it was very successful, successful, or not successful. Users can filter protocols based on whether it was successful, the tissue and organism studied, and the details of five different tissue clearing steps.

## Materials and methods

### Tissue clearing survey

The tissue clearing survey was developed by the 10-member BRAIN Initiative 3D Microscopy Working Group (WG) (https://doryworkspace.org/WorkingGroupRoster) between November 2019 and January 2020. The goal of the WG was to establish standards to promote data sharing within the research community. The WG prioritized two approaches for developing standards: (1) to develop standard metadata, which has been described elsewhere (Ropelewski et al., 2021); and (2) to investigate the feasibility of recommending standard protocols for tissue clearing. Because of the rapidly evolving nature of the field, the WG agreed that it was premature to recommend one or even a group of tissue clearing protocols for use by all investigators. In fact, the proliferation of new methods and numerous experimental conditions that must be considered makes it challenging for investigators to select and optimize a tissue clearing protocol for their own experiments. To address this problem, the WG agreed that a platform was needed to help investigators review and compare protocols. Furthermore, this platform should not be limited to brain studies because the same problems apply to other experiments in other tissues.

WG members reviewed the literature and consulted with colleagues to identify the most widely used tissue clearing techniques and key experimental parameters that impact selection of a protocol and tissue clearing success or failure. Based on this input, the subgroup developed a table of commonly used tissue clearing protocols as a framework for gathering and comparing methods between laboratories. The table included protocol-specific details for five generalized steps commonly used in tissue clearing (Richardson and Lichtman, 2017; Weiss and Huisken, 2021). The table also included a list of experimental constraints that impact selection (e.g., time, cost, use in literature) and success (e.g., specimen, tissue, type of analysis, available equipment).

**FIGURE 1:**
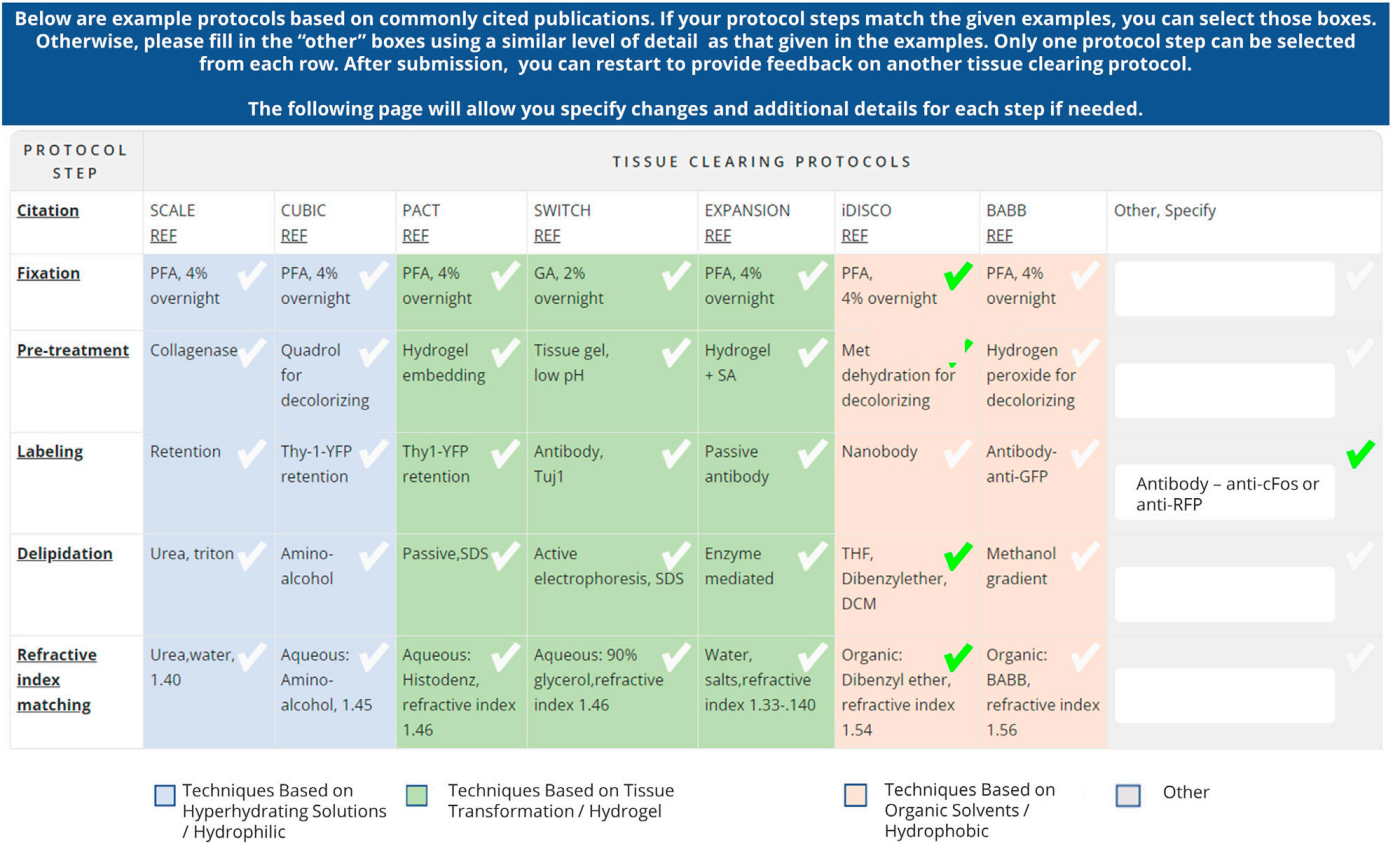
Table of commonly used tissue clearing protocols included in the Tissue Clearing Survey (https://doryworkspace.org/tissue_clearing_feedback). Respondents identified the tissue clearing protocols used by checking boxes in the table or by entering details in the “Other, specify” column. The table demonstrates an example tissue clearing protocol response based on iDISCO with a modification to the labeling step indicated in the “Other, Specify” column.

The table was then embedded in a larger survey to gather feedback on tissue clearing protocol experiments (https://doryworkspace.org/tissue_clearing_feedback). The first part of the survey captured experimental details (e.g., specimen type, tissue type, feature of interest), why the protocol was chosen, whether the protocol was successful, and reasons why an experiment may have failed (if applicable). The survey then presented the table of commonly used published protocols (Figure 1). The name of each published protocol was listed in column headers while five generalized steps were shown in the row headers. Each cell briefly summarized some of the published details for that protocol for that step. The table also classified the individual protocols according to mode of action (techniques based on hyperhydrating solutions (hydrophilic), tissue transformation (hydrogel), and organic solvents (hydrophobic). Respondents reported the tissue clearing protocol they used by either selecting a cell from a published protocol or specifying modifications using the “other” boxes. Respondents were asked to provide a citation (e.g., publication reference, DOI) for any details in the “other” boxes because these represented modifications to one of the protocols listed or use of a newer protocol not included in the table. Subsequent survey questions captured additional experimental details including type of microscope used, image analysis, and job title of the respondent.

The tissue clearing survey was emailed to investigators who were either 1) experts nominated by the BRAIN Initiative 3D Microscopy Working Group 2) Principal Investigators of recent NIH-funded grants investigating relevant tissue clearing or microscopy techniques (identified using key word searches of abstracts available from the NIH RePORTER (https://reporter.nih.gov/advanced-search) and the BRAIN Initiative websites (https://braininitiative.nih.gov/) or 3) corresponding and/or senior authors of recent publications reporting on relevant tissue clearing or microscopy techniques (identified using keyword searches of abstracts available on pubmed.gov). The tissue clearing survey email described the goals of the project and provided a link to the survey on a private page of the DORy website (https://doryworkspace.org/tissue_clearing_feedback). The initial survey was open between November 2020 and January 2021 and yielded 29 responses. In an effort to increase response rate, a second email was sent to the same list of experts in October 2021 and the survey remained open until January 2022. The second outreach effort yielded 11 more responses.

### Analysis of tissue clearing survey results

The results of the tissue clearing survey were downloaded from the DORy website in CSV format and imported into Microsoft Excel^®^. Each unique entry describing a tissue clearing protocol was recorded in a separate row. For each entry, the response for each survey question is recorded in a separate column (see complete dataset here: https://doryworkspace.org/doryviz).

**FIGURE 2:**
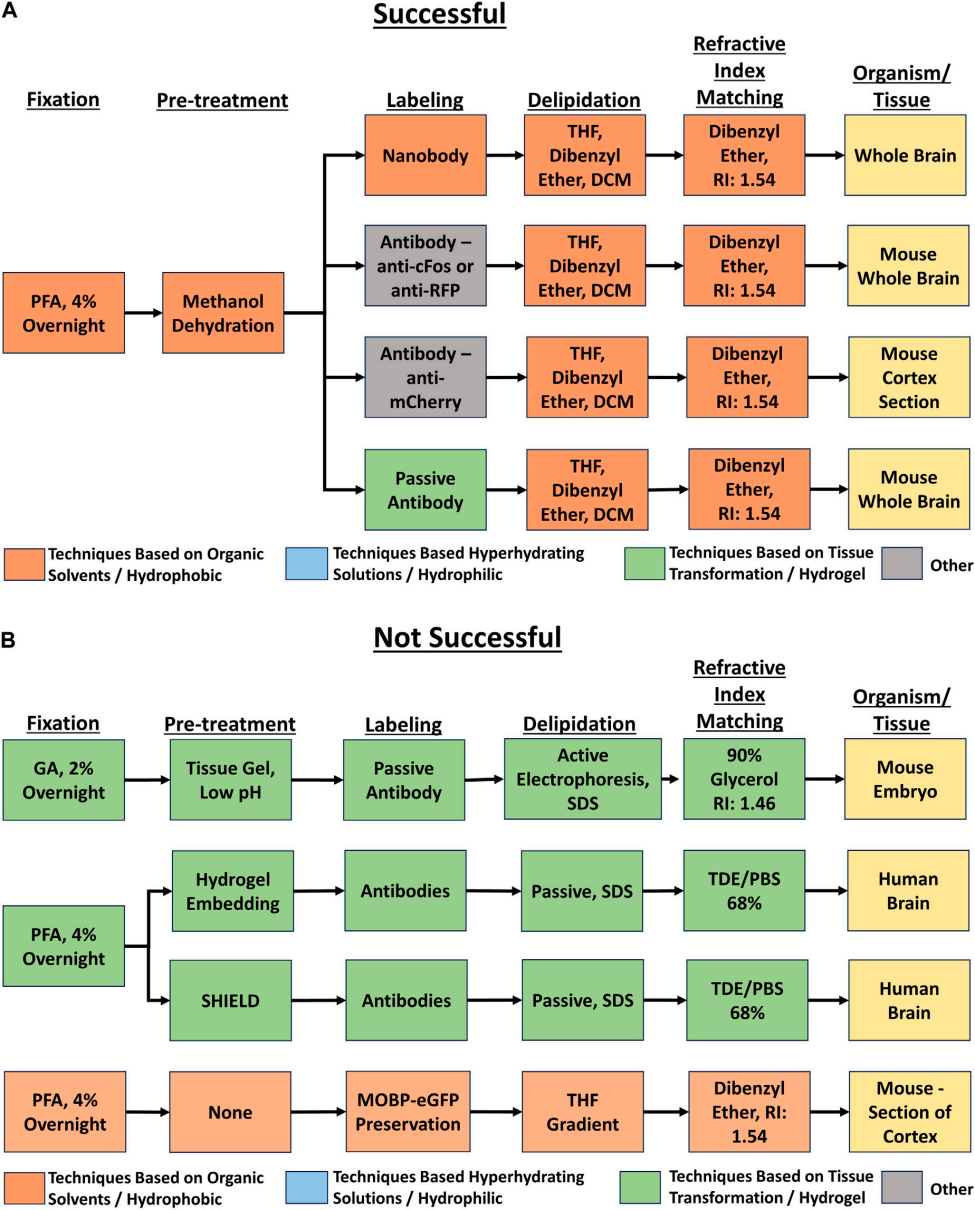
Example flow charts created from reported tissue clearing protocols. **(A)** Example flow charts of successful tissue-clearing protocols. The tissue cleared is indicated in the right-most box. **(B)** Example flow charts of not successful tissue-clearing protocols. The tissue cleared is indicated in the right-most box.

Each unique tissue clearing protocol was diagramed as a flow chart using Lucidchart software (Figure 2). Each flow chart included reported details for five major tissue clearing steps) and the organism and tissue being cleared. Each protocol flow chart was organized according to whether the respondent indicated the protocol was very successful, successful, or not successful.

### Development of T-CLEARE

Each flow chart was converted to a JavaScript Object Notation (JSON) tree data structure where the five different protocol steps were mapped to an arrays of key:value pairs [e.g., Fixation (*key*): PFA 4% overnight (*value*)]. The JSON structure also contained some attributes to define the visualization part of the chart such as protocol step images, orientation of the nodes, or animation.

The individual JSON arrays were connected to a top-level parent “Protocol” node. The three children of the Protocol node are the key:value pairs indicating whether the protocols were “Very Successful,” “Successful,” and “Not Successful.” The fixation step is the child of protocol success, and each subsequent protocol step is the child of the previous protocol step (e.g., the delipidation step is the child of the fixation step). The tissue being cleared is the child of the refractive index matching protocol step (i.e., the leaf node).

The T-CLEARE presentation layer was implemented using pure JavaScript (JS) and the Treant.js (https://fperucic.github.io/treant-js/) open-source visualization library. The code included the jquery.min.js library to access and manipulate HTML elements. A custom JS library (dory-custom.js) was written to support the filters. CSS was used for styling. The T-CLEARE presentation layer was integrated into the Drupal-8 based DORy website (https://doryworkspace.org/doryviz) by packaging the JS and CSS files along with the JSON tree structure into a custom Drupal 8 module. T-CLEARE was tested with Chrome Version 101.0.4951.64 and is compatible with any browser (e.g., Firefox, Safari, and Microsoft Edge).

### Availability

A standalone implementation of T-CLEARE is available from our GitHub repository (https://github.com/Defining-Our-Research-Methodology-DORy/T-CLEARE). The lightweight T-CLEARE application can be run in any browser as a standalone client or integrated and deployed in other platforms.

## Results

### T-CLEARE

**FIGURE 3:**
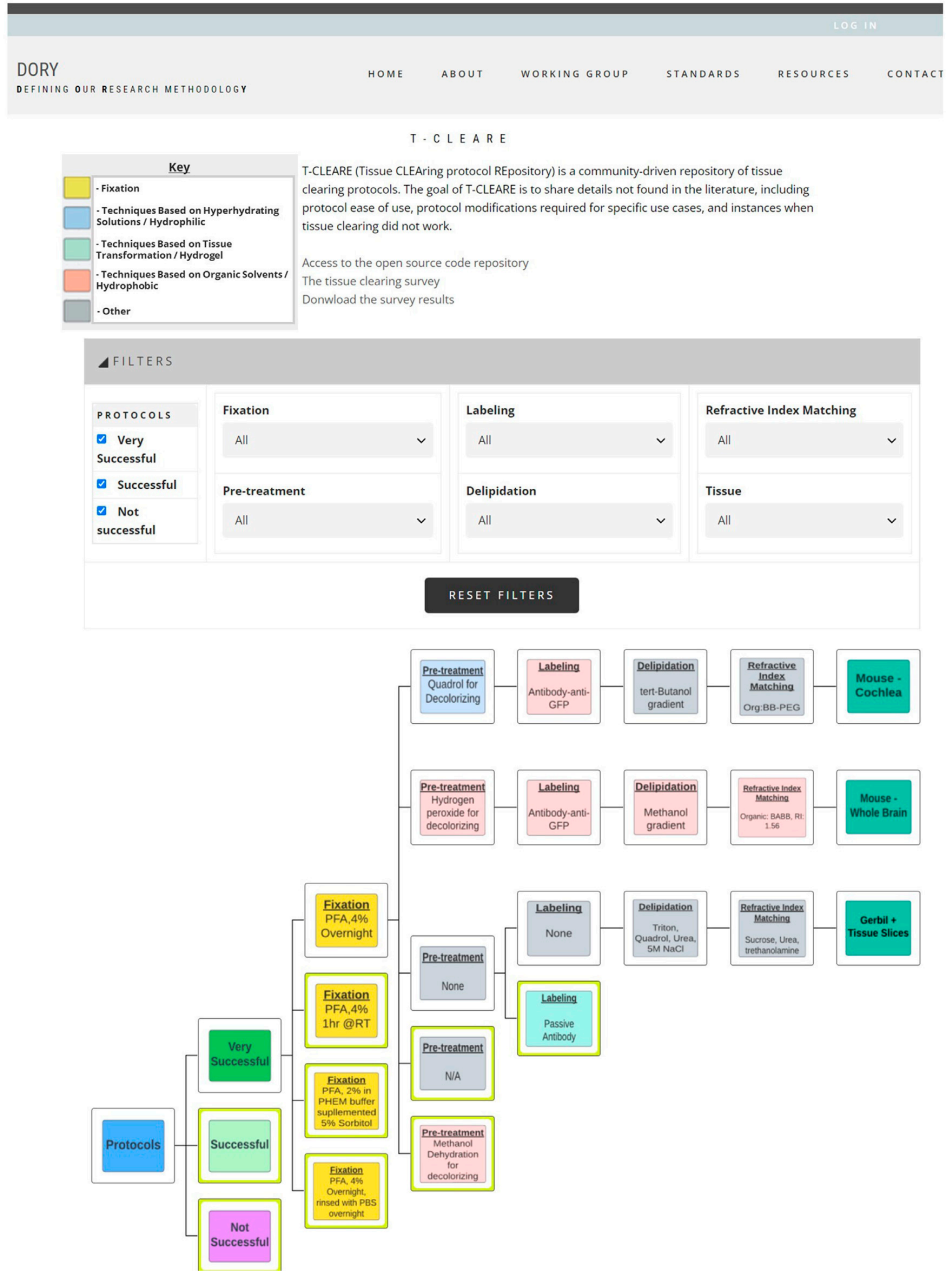
Screenshot of T-CLEARE user interface. Boxes in light blue, aqua, and light red indicate whether the mode of action for that step is based on hyperhydrating solutions/hydrophilic, tissue transformation/hydrogel, and organic solvents/hydrophobic, respectively. Yellow boxes indicate the fixation step that can be classifed according to more than one mode of action. Gray boxes indicate the respondent entered text in the “Other, specify” in the survey table.

T-CLEARE displays a tree diagram including each tissue clearing protocol from the survey (Figure 3; https://doryworkspace.org/doryviz). When a user first opens T-CLEARE, the tree diagram displays four boxes. The “Protocols” box includes all flow diagrams for the reported tissue clearing protocols. The next level to the right includes three boxes that organize the flow diagrams according to whether the protocols were “Very Successful,” “Successful,” or “Not Successful.” Clicking these three boxes will display the associated tissue clearing protocol flow diagrams. The color of the box in the flow diagrams indicates whether the protocol step is associated with a hyperhydrating/hydrophilic (blue), tissue transformation/hydrogel (aqua), or organic solvent/hydrophobic (pink) based class of tissue clearing technique. Gray boxes indicate that the survey respondent used an “Other, specify” box to enter details for a protocol step. The fixation steps are yellow because these methods (e.g., PFA 4%, overnight) are shared among the classes of techniques.

The user can stratify the list of tissue clearing flow diagrams displayed in the T-CLEARE using the filters at the top of the page. Available filters include level of success (e.g., very successful, successful, not successful), details for each of the five major steps of the protocol) and organism and tissue type (e.g., mouse–whole brain; mouse–cochlea). These filters can help users determine how successful a method, or group of methods, is for a given tissue. For example, users can select “successful” protocols that follow hyperhydrating/hydrophilic principles for fixation, pretreatment, labeling, and refractive index matching in “mouse brain.”

## Discussion

The goal of the T-CLEARE pilot is to provide a high-level summary of user’s experiences optimizing tissue clearing protocols and to catalog the expanding combinations of tissue types, antibodies/labels, and clearing protocols. T-CLEARE is not intended to enable replication, but to help users prioritize which tissue clearing protocols to try for a given experiment. The T-CLEARE survey sought to capture details not commonly reported in the literature, including negative results and protocol modifications needed for a specific use case. The survey was also structured to support development of visualization tools to help users quickly compare among tissue clearing protocols.

**FIGURE 4:**
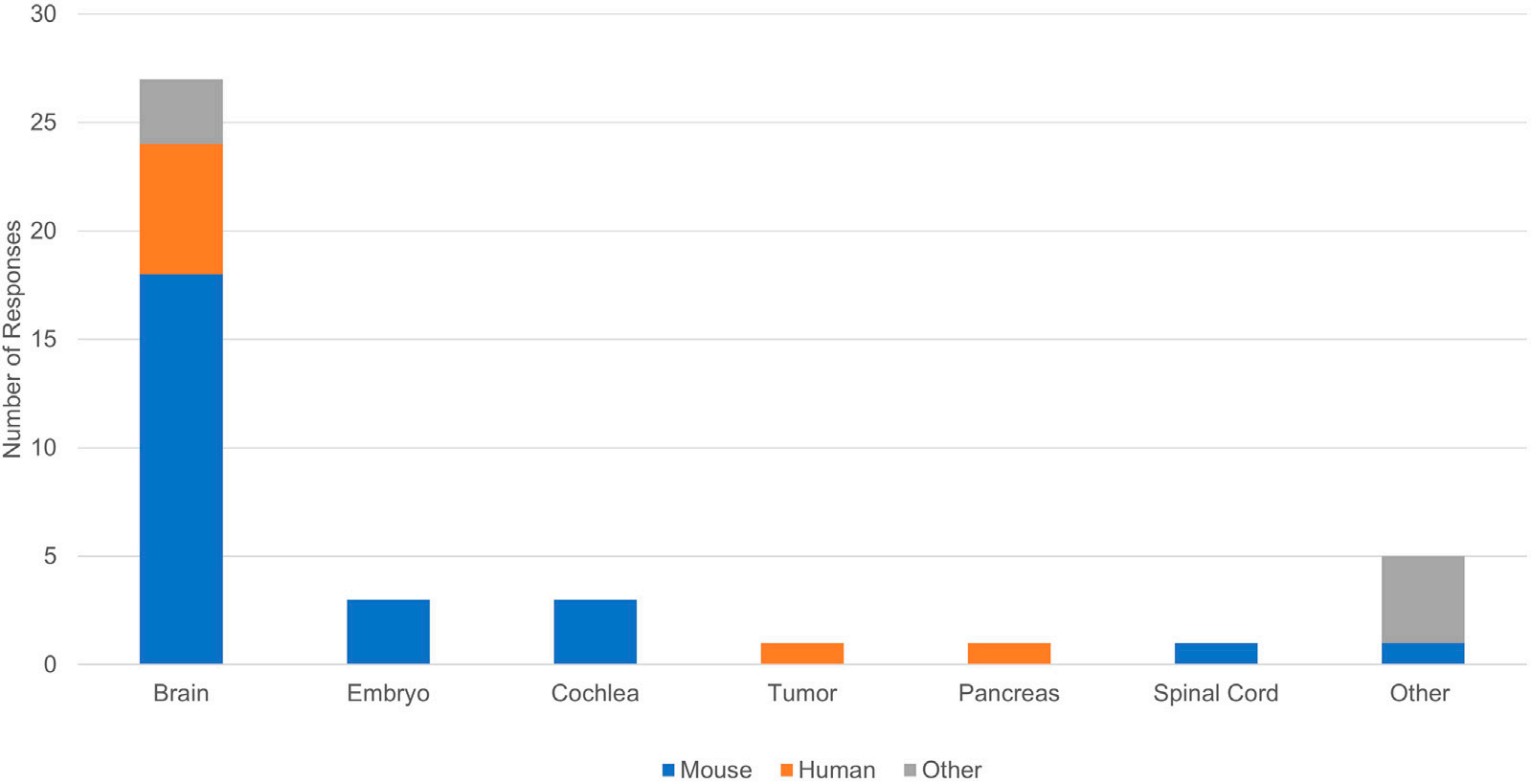
Organisms and tissues in reported tissue clearing protocols.

**FIGURE 5:**
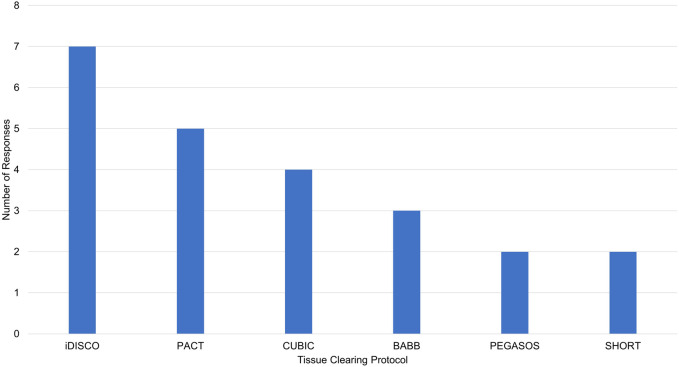
Reported tissue clearing protocols used without modification. iDISCO, immunolabeling-enabled three-Dimensional Imaging of Solvent-Cleared Organs (Renier et al., 2014), PACT, PAssive Clarity Technique (Yang et al., 2014), CUBIC, Clear, Unobstructed Brain Imaging Cocktails and Computational analysis (Tainaka et al., 2014), BABB, Benzyl Alcohol/Benzyl Benzoate (Dodt et al., 2007), PEGASOS, polyethylene glycol (PEG)-associated solvent system (Jing et al., 2018), SHORT, SWITCH - H2O2 - antigen Retrieval (Pesce et al., 2022).

**FIGURE 6:**
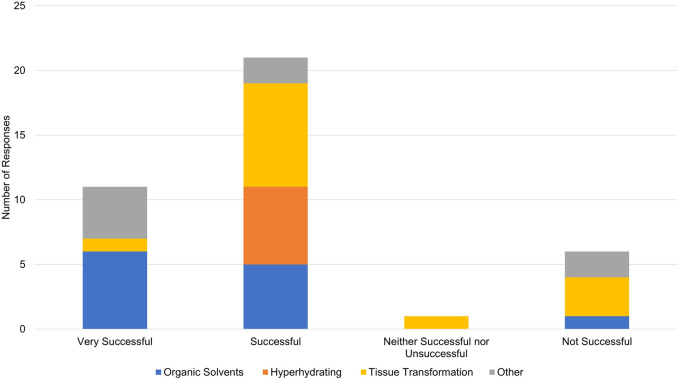
Overall success in reported tissue clearing protocols. Individual protocols in responses have been grouped according to mode of action (Organic Solvents/Hydrophobic, Hyperhydrating Solutions/Hydrophilic, Tissue Transformation/Hydrogel, Other).

Limitations of our tissue clearing survey results include a relatively small number of responses, write-in responses which can be challenging to categorize, and over-representation of experiments in mouse brain (Figure 4). Despite limitations, our results support previously identified trends. iDISCO (Renier et al., 2014) was the most frequently cited protocol in our survey (Figure 5), consistent with it being the most popular method reported in the literature (Costantini et al., 2019). Fifteen of the 41 responses indicated that they made modifications to a published tissue clearing protocol supporting the claim that protocols may require optimization when applied to new experimental parameters (Richardson et al., 2021; Weiss and Huisken, 2021). Our survey results support the assertion that there is not a single best technique that can be used successfully across all experimental conditions (Berman et al., 2000; Costantini et al., 2019). Figure 6 shows reported success of protocols when grouped according to mode of action (e.g., hyperhydrating/hydrophilic techniques, tissue transformation/hydrogel techniques, organic solvent/hydrophobic techniques). While six of the 11 tissue clearing protocols that were “Very Successful” were based on organic solvent/hydrophobic techniques, an examination of the 21 “successful” tissue clearing protocols reveals relatively even distribution among hyperhydrating/hydrophilic (six protocols), tissue transformation/hydrogel (eight protocols), and organic solvent/hydrophobic techniques (five protocols).

While a strength of our pilot is capturing negative results, they should be interpreted with caution. Of the six protocols that were “Not Successful”, four were based on tissue transformation/hydrogel techniques, one was based on organic solvent/hydrophobic techniques [FDISCO (Qi et al., 2019)], and one was classified as “other.” This should not be interpreted to mean that techniques based on tissue transformation/hydrogel are not effective. In the survey, Tissue transformation/hydrogel techniques were most often reported (14 responses) followed by organic solvents/hydrophobic (12 responses) and hyperhydrating/hydrophilic techniques (6 responses). Additionally, Figure 6 demonstrates that tissue transformation/hydrogel techniques account for nine of the 32 “Successful” and “Very Successful” responses. While the distribution of tissue transformation/hydrogel techniques across success categories is slightly skewed, this could be explained by the low number of survey responses overall.

It is important to note that the terms “Very Successful”, “Successful, and “Not Successful” are subjective. In particular, “Not Successful” may encompass a variety of reasons why an experiment failed (e.g., the sample could have been successfully cleared, but not at sufficient resolution to answer a specific question). For example, one of the “Not Successful” tissue transformation/hydrogel protocols used the SHIELD (Park et al., 2018) protocol to clear different brain sections from adult humans for 3 days rendering of cells. The respondent noted lack of success was due to antibodies not penetrating the tissue instead of insufficient clearing. Similar details for all experiments can be reviewed by downloading the survey results from the website.

**FIGURE 7:**
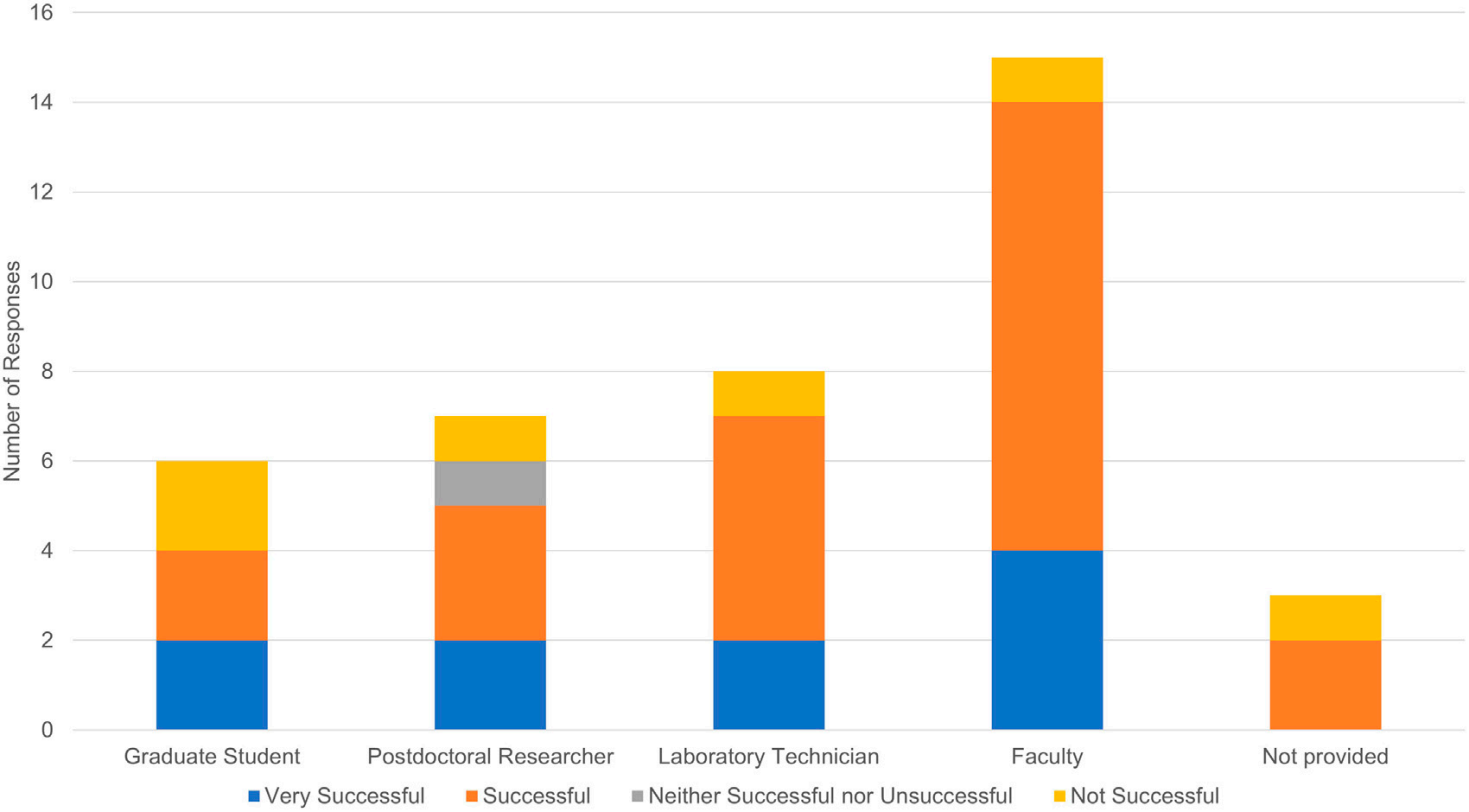
Reported tissue clearing success by respondent job title.

Another potential explanation for unsuccessful tissue clearing experiments is lack of experience and technical proficiency with a given protocol. Our survey did not ask respondents to describe their expertise and whether they had performed benchmark experiments. While not equivalent, respondents did indicate career stage and job title which may provide additional context (see Figure 7). Fifteen respondents indicated they were faculty, seven were postdoctoral researchers, eight were laboratory technicians, six were graduate students, and three did not provide a job title. Unsuccessful experiments were reported by one faculty member, one postdoctoral researcher, two laboratory technicians, and one “other”.

The flowcharts on T-CLEARE website provide a quick and easy way to visualize and review the survey results on a single page. However, the flowcharts are necessarily high-level and do not include many of the reported details. Users are encouraged to download the raw survey results from the website which provide additional details including overarching comments, rationale and supporting references supporting deviations from published methods referenced in the table, and reasons why a protocol may be classified as unsuccessful. While the survey did accept write-in responses to capture newer technologies with associated references, the email blast was performed in early 2021. Due to rapid innovations taking place in the field, the flowcharts may not reflect the current state of the art.

There are several ways to increase the utility of T-CLEARE. The most obvious is to gather additional input from the scientific community. To this end, the tissue clearing survey will remain open and accessible from the DORy website (https://doryworkspace.org/tissue_clearing_feedback) for the community to report results of additional tissue clearing experiments. There could also be updates to the survey to ask about technical proficiency with a given protocol and to gather feedback on new protocols (e.g., expansion microscopy). It may also be helpful to include links to existing public forums or create forum on T-CLEARE where users can post comments and questions linked to specific tissue clearing protocols in the flowcharts.

Future versions of T-CLEARE could automate the flow of the data from the survey to the user interface. Although the current lightweight JS/JSON implementation of T-CLEARE is modular (can be run standalone in any browser or easily integrated into other platforms) and scalable (additional protocols can be integrated as new JSON arrays), analyzing the survey results, curating the protocols flow diagrams, and creating images displayed in the nodes are manual steps. Future versions could include a MySQL database to capture and store the survey responses and algorithms that dynamically process new responses, generate the JSON arrays and images, and add them to the tree. Updates to the T-CLEARE user interface could include additional filters (e.g., microscope type used, type of analysis, level of analysis) and detailed comments from respondents. These comments, which provide details about protocol modifications, optimization, references, and why an experiment may have failed, are difficult to classify and are currently only available in the raw Excel data file. Future T-CLEARE features could link to full text versions of tissue clearing protocols and resulting images in data repositories.

In summary, T-CLEARE is a novel, web-based pilot repository of tissue clearing protocols to help the community share, find, and evaluate available tissue clearing protocols in real time. The community is encouraged to provide comments and suggestions using the feedback tools on the T-CLEARE GitHUB repository pages. As more information is added, we hope that T-CLEARE can help the community coalesce around reporting standards and standard tissue clearing protocols. These community standards will help ensure consistent data collection and reporting, improve data interpretation, and facilitate data sharing among the scientific community. Finally, T-CLEARE lays the groundwork to develop a knowledgebase of the innumerable combinations of antibodies, tissue types, and clearing protocols in a sortable, searchable manner. Such a database could also be used with Artificial Intelligence/Machine Learning algorithms to predict which combinations of steps are likely to be successful for a given experiment.

## Data Availability

The datasets presented in this study can be found in online repositories. The names of the repository/repositories and accession number(s) can be found below: https://github.com/Defining-Our-Research-Methodology-DORy/T-CLEARE, https://doryworkspace.org/doryviz.
